# Calibration-Free, Seconds-Resolved In Vivo Molecular Measurements using Fourier-Transform Impedance Spectroscopy Interrogation of Electrochemical Aptamer Sensors

**DOI:** 10.1021/acssensors.3c00632

**Published:** 2023-08-16

**Authors:** Brian Roehrich, Kaylyn K. Leung, Julian Gerson, Tod E. Kippin, Kevin W. Plaxco, Lior Sepunaru

**Affiliations:** † Department of Chemistry and Biochemistry, University of California Santa Barbara, Santa Barbara, California 93106, United States; ‡ Department of Psychological and Brain Sciences, University of California, Santa Barbara, California 93106, United States; § Department of Molecular Cellular and Developmental Biology, University of California, Santa Barbara, California 93106,United States; ∥ Center for Bioengineering, University of California Santa Barbara, Santa Barbara, California 93106, United States

**Keywords:** electrochemical sensor, electrochemical impedance, aptamer sensor, in vivo, fast-Fourier transform

## Abstract

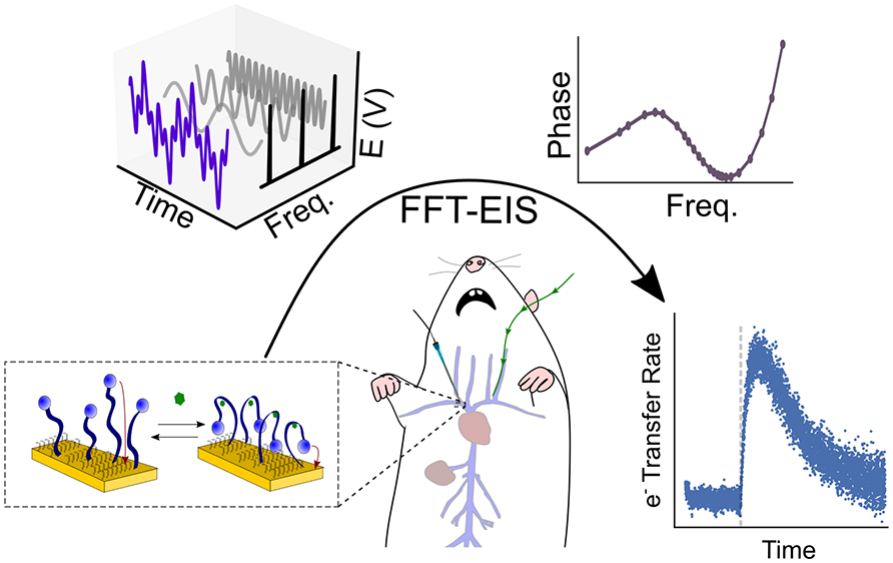

Electrochemical aptamer-based (EAB) sensors are capable of measuring the concentrations of specific molecules in vivo, in real time, and with a few-second time resolution. For their signal transduction mechanism, these sensors utilize a binding-induced conformational change in their target-recognizing, redox-reporter-modified aptamer to alter the rate of electron transfer between the reporter and the supporting electrode. While a variety of voltammetric techniques have been used to monitor this change in kinetics, they suffer from various drawbacks, including time resolution limited to several seconds and sensor-to-sensor variation that requires calibration to remove. Here, however, we show that the use of fast Fourier transform electrochemical impedance spectroscopy (FFT-EIS) to interrogate EAB sensors leads to improved (here better than 2 s) time resolution and calibration-free operation, even when such sensors are deployed in vivo. To showcase these benefits, we demonstrate the approach’s ability to perform real-time molecular measurements in the veins of living rats.

The availability of sensors able to measure the concentrations of specific molecules in the body in real time would revolutionize the monitoring of health and the diagnosis and treatment of disease. By providing a real-time window into plasma drug concentrations, for example, such an advance would significantly improve the individualization of pharmacological treatments.^1^ Likewise, the highly time-resolved measurement of metabolites, neurotransmitters, and hormones enabled by such sensors could significantly advance our understanding of physiology. Motivated by this promise, we and others have been developing electrochemical aptamer-based (EAB) sensors.^2−4,5,6,7^ EAB sensors are composed of a gold electrode on which a submonolayer of target-recognizing, redox reporter-modified, nucleic acid aptamers are deposited via thiol-on-gold self-assembled monolayer formation.^6^ Introduction of the specific target molecule triggers a conformational change in this aptamer, altering the distance between the redox reporter and the electrode surface and changing, in turn, the rate of electron transfer (*k*_et_) from the attached redox reporter (Figure 1). This change in *k*_et_, which, to date, has been monitored using a range of electrochemical approaches, reports on the target concentration in real-time without the addition of exogenous reagents. Of note, this signal transduction mechanism does not rely on the chemical transformation of the target, rendering the approach general. Consistent with this, EAB sensors have been shown to support the high-frequency, real-time measurement of multiple drugs,^7−9,10^ metabolites,^11,12^ and protein biomarkers,^3,13,14^ both in vitro and in vivo.

**Figure 1:**
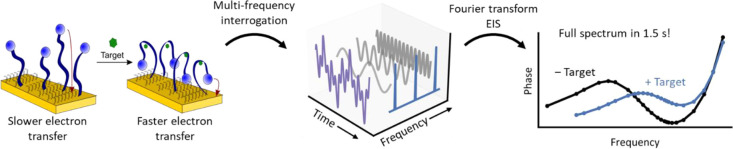
(left) EAB sensors rely on the target-induced conformational change of a DNA aptamer to produce a change in electron transfer kinetics. Upon target binding, the redox label is brought closer to the electrode, increasing the electron transfer rate constant. (middle) The resulting change in the electron transfer rate can be rapidly measured using fast Fourier transform electrochemical impedance spectroscopy (FFT-EIS). In this, sinusoidal oscillations of several different frequencies are summed, and the resultant waveform is applied as an AC voltage perturbation to the working electrode of the EAB sensor (right). Fourier transform of the applied, multifrequency, voltage, and the current response yields a full impedance spectrum rapidly enough to support real-time sensor interrogation with time resolution limited (here to less than 2 s) by the lowest frequency sinusoidal perturbation employed.

A variety of electrochemical interrogation methods, including cyclic voltammetry (CV),^15^ chronoamperometry,^16^ AC voltammetry,^3,4,17^ square wave voltammetry (SWV),^7,18^ and intermittent pulse amperometry^19^ have been employed in the interrogation of EAB sensors. While each of these has advantages and disadvantages,^20^ SWV has seen the most widespread use in vivo. This is because measuring sequential square wave voltammograms at two different frequencies enables drift correction in an approach called kinetic differential measurements (KDM).^21^ Using KDM, which employs the difference between SWV measurements taken at two frequencies to subtractively remove drift, we have achieved multihour measurements of multiple target molecules in situ in the veins of living animals.^7,8,22^ The resulting need to collect two square wave voltammograms per measurement point, however, reduces the time resolution of such measurements to, typically, 6 to 22 s.^6,7,9,11,22^ Because it relies on the monitoring of peak currents, SWV-based interrogation is also susceptible to sensor-to-sensor fabrication variation arising from differences in the number of methylene-blue-modified aptamers placed on each. Because of this, sensors employing a SWV must be individually calibrated before use.

Here, we demonstrate electrochemical impedance spectroscopy (EIS) as an alternate EAB sensor interrogation method, one that does not require calibration and achieves superior time resolution. EIS is widely used in biosensing due to the depth of information it provides on the electrode–electrolyte interface.^23−25,26^ In this approach, the impedance between the working electrode and the counter electrode is measured as a function of frequency. At higher frequencies, this impedance informs on rapid processes such as the formation of the electrochemical double layer. Impedances measured at lower frequencies, in contrast, are typically associated with slower processes, such as electron transfer, adsorption and intercalation events, as well as mass transport.^27−29^ Despite the broad insights EIS can provide, its adaptation to the interrogation of EAB sensors has had relatively little investigation.^30,31^ For example, in the broadest study to date, impedimetric phase shift at a single frequency (rather than the collection of an entire impedance spectrum) was used to monitor changing target concentrations in real-time.^31^ However, while such phase monitoring achieves exceptional, ∼ 300 ms, time resolution; this single-frequency approach required the calibration of individual sensors and was not demonstrated to work in vivo. Here, we have taken a different approach to employing EIS in the interrogation of EAB sensors. Specifically, we have used fast Fourier transform electrochemical impedance spectroscopy (FFT-EIS) to simultaneously measure the impedance of in vivo EAB sensors at multiple frequencies, yielding both rapid time resolution and the depth of information contained in a full impedance spectrum.^32−34,35^ With this, we can estimate *k*_et_ – and from that, the target concentration–every few seconds (here less than 2 s), providing a method of in vivo sensor interrogation that is both more highly time-resolved and calibration-free.

## Results and Discussion

In EIS, a sinusoidal oscillating voltage on top of a set DC bias is applied to the working electrode, and the (sinusoidal) current response is recorded.^29^ The impedance, *Z*, at a particular frequency ω is defined as the ratio between voltage and current at that frequency (eq 1),^24^ with the “lag” between the voltage perturbation and the current response quantified as the phase shift ϕ.
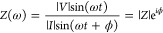
1Here, |*V*| and |*I*| are the amplitudes of the voltage and current, respectively; ω is the frequency; *i* is the square root of −1, and |*Z*| is the magnitude of the impedance. The primary benefit of EIS (and its label of “spectroscopy”) arises from the measurement of *Z* across a wide range of frequencies (e.g., milli- to kilohertz). Specifically, frequency-dependent impedance measurements can be used to characterize processes ranging from the rapid charging of the electric double layer at high frequencies to electron transfer reactions and molecular diffusion occurring on much longer time scales.^27,36^

In applications such as ours–the real-time measurement of specific molecules in the living body– a limitation of EIS is that its time resolution is typically rather poor. Specifically, with traditional, “frequency-sweep” EIS, each frequency, *f,* interrogated requires at least a *1/f* measurement time. Given this, the measurement of spectra down to frequencies of order 1 Hz requires total acquisition times of at least ten seconds. FFT-EIS, however, retains the information contained in the entire frequency range while significantly decreasing this acquisition time. It does so by measuring the impedance at all frequencies simultaneously.^32,34,35,37^ In our implementation of FFT-EIS, the applied voltage perturbation is a superposition of 18 sine waves spanning the desired frequency range. Due to the (approximate) linearity of electrochemical systems over small voltage changes,^27^ the resulting current response is a superposition of the current response at each applied frequency. A Fourier transform of the recorded voltage and current data thus yields an impedance spectrum.^33,35^ Using this approach, we can collect a complete impedance spectrum on the time scale defined by the slowest applied frequency (*t*_spectrum_ ≥ 1/*f*_min_). For example, an impedance spectrum spanning 18 frequencies from 1 Hz to 1 kHz, which would require ∼23 s to collect on our potentiostat (Autolab PGStat128N) using traditional EIS, can be measured in 1.8 s using our FFT-EIS implementation.

**Figure 2:**
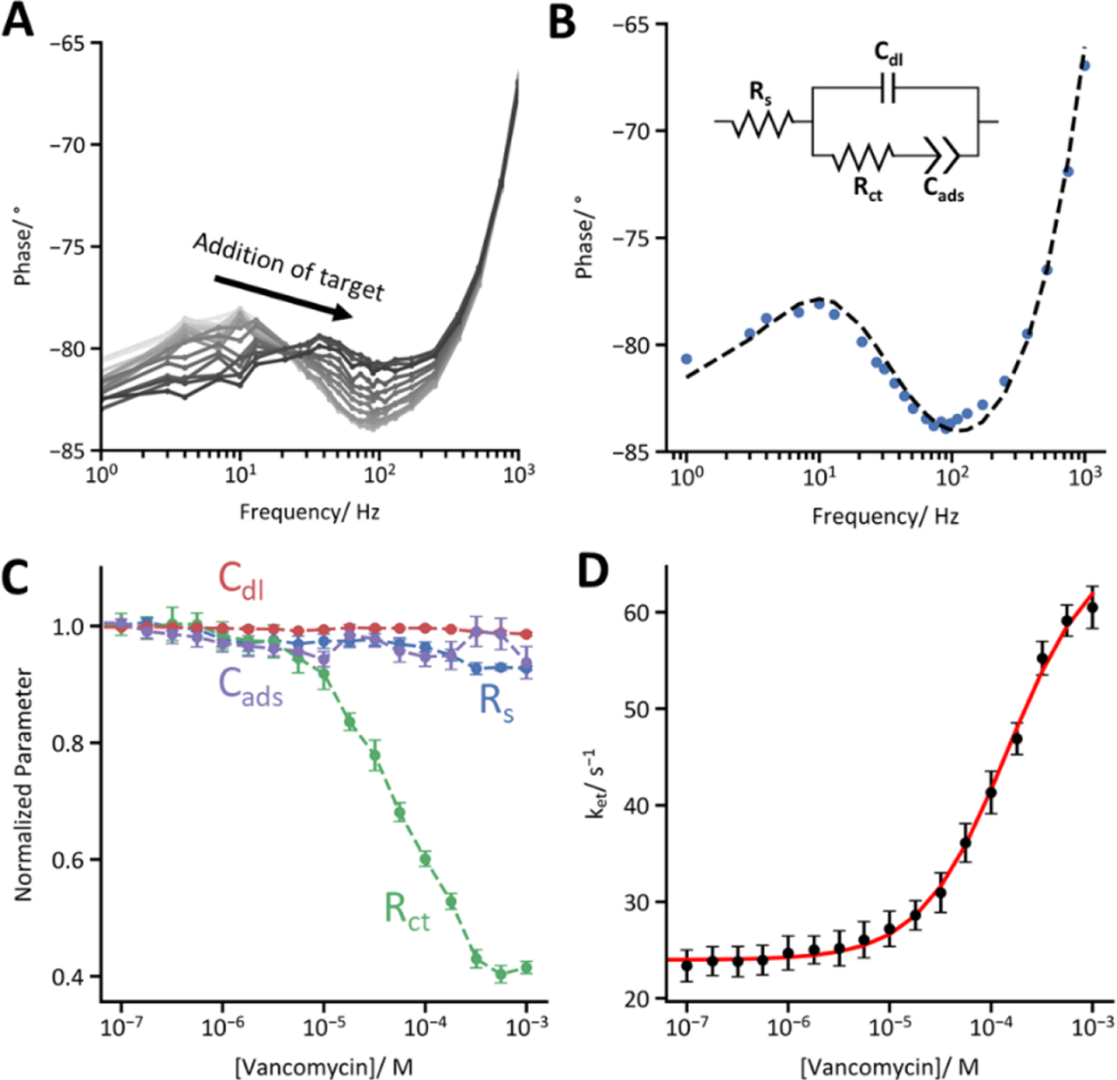
When used to interrogate an EAB sensor, FFT-EIS is able to rapidly correlate electrochemical impedance to the concentration of a target molecule. (A) A vancomycin-detecting EAB sensor was immersed in whole bovine blood at 37 °*C* and exposed to increasing quantities of its target. This induced a conformational change in the aptamer that caused a shift in the impedance spectrum, as resolved by FFT-EIS. (B) To analyze such FFT-EIS data we employ equivalent circuit modeling, using a circuit model comprised of resistors representing the bulk solution resistance (*R*_s_) and the Faradaic electron transfer between the electrode and the methylene blue moieties (*R*_ct_), as well as capacitors representing the electrochemical double layer (*C*_dl_) and the pseudocapacitance between the electrode surface and the surface-bound methylene blue (*C*_ads_).^38^ We calculated the transfer function of this circuit using Kirchhoff’s laws and used it to fit our experimentally measured data. The data presented here were collected from a vancomycin-detecting sensor immersed in whole bovine blood at 37 °C in the absence of vancomycin. (C) Challenging the EAB sensor with increasing concentrations of its vancomycin target reveals that increasing target concentration predominantly impacts (here, decreases) *R*_ct_. This is because a low electron transfer resistance corresponds to a high electron transfer rate constant, corresponding to the target-bound state of the aptamer (in this figure, the error bars represent standard deviations across four independently fabricated and interrogated sensors). (D) Electron transfer rate *k*_et_ can be approximated from *R*_ct_ and *C*_ads_ using eq 4. The resulting binding curve fits a Hill–Langmuir isotherm with a dissociation constant *K*_D_ = 144 ± 31 μM (the latter reflects estimated 95% confidence intervals).

The impedimetric properties of EAB sensors, which are sensitive to target concentration, can be rapidly measured using FFT-EIS to enable highly time-resolved molecular measurements.^8,39,40^ To demonstrate this, we used a previously reported aptamer to fabricate an EAB sensor targeting the antibiotic vancomycin.^8^ We then applied the half-wave potential (*E*_1/2_) of the sensor’s methylene blue redox reporter as the DC bias (prior to each experiment, we use cyclic voltammetry to determine *E*_1/2_, which is typically around −0.285 V versus Ag/AgCl; Figure SI1). We then used FFT-EIS to record impedance spectra as the sensor was immersed in whole bovine blood at 37 °C. When the spectrum obtained in the absence of target molecule is displayed as a Bode plot (phase versus frequency;^29^Figure 2A, black trace), a local maximum is observed around 10 Hz, reflecting the electron transfer rate between the methylene blue and the electrode surface.^31^ As expected (given that the rate of electron transfer between the methylene blue and the electrode increases upon target binding),^31^ this peak steadily shifts to higher frequencies when the sensor is exposed to increasing vancomycin concentrations. To determine the origin of this concentration-dependent shift, we applied equivalent circuit modeling^23,24^ using a simple, four-element equivalent circuit that has previously been used to represent the surface-tethered redox species seen in EAB sensors (Figure 2B).^31,38,41^ This circuit consists of a resistor, modeling bulk solution resistance (*R*_s_), that is in series with the three other components: a capacitor, representing interfacial double-layer capacitance (*C*_dl_), in parallel with a resistor, representing the Faradaic charge transfer resistance (*R*_ct_), and a capacitor (*C*_ads_), representing the surface-attached methylene blues. To better account for the rough, nonideal surface of the EAB sensor, the latter is modeled as a constant phase element, rather than a true capacitor.^42^ This model fits our FFT-EIS data quite well (χ^2^ ∼ 0.015, Figure 2B), suggesting that this four-component circuit is an adequate description of the physics of our sensors.

The components of the equivalent circuit behave as expected in response to increased vancomycin concentrations. *R*_s_, *C*_dl_, and *C*_ads_, for example, are effectively independent of vancomycin concentration. *R*_ct_, in contrast, decreases with an increasing vancomycin concentration (Figure 2C). This presumably arises due to the increased rate of electron transfer between the electrode and the bound, folded aptamer, as *R*_ct_ is inversely proportional to *k*_et_:^38^
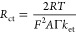
2Here, *R* is the gas constant, *T* is temperature, *F* is Faraday’s constant, *A* is the electrochemical surface area of the working electrode, and Γ is the surface coverage of the redox-active molecule. Other than *k*_et_, all of these variables are constant during a given experiment, and thus the decrease in *R*_ct_ is entirely attributed to an increase in *k*_et_. Since, in turn, *C*_ads_ is given by^38^
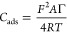
3*k*_et_ can be calculated from the *R*_ct_ and *C*_ads_ as^38^
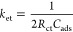
4

Given that the rate of electron transfer from the methylene blue depends on whether the aptamer is target bound, *k*_et_ should trace a Langmuir–Hill isotherm when plotted versus the vancomycin concentration. As expected, it does (Figure 2D). The resulting monotonic relationship can be used to convert *k*_et_ to estimates of vancomycin concentration in a manner that is calibration-free. Specifically, *k*_et_ is independent of the number of surface-bound methylene blue species. It thus is independent of important sensor-to-sensor sources of fabrication variability, such as changes in the surface area of the electrode (Figure SI2), or the aptamer packing density, which would change the number of methylene blues and thus the absolute Faradaic current. Because of this, a *k*_et_ versus vancomycin concentration calibration curve measured for a single sensor can be applied to all other sensors utilizing the same aptamer, obviating the need to calibrate each individual sensor.

**Figure 3:**
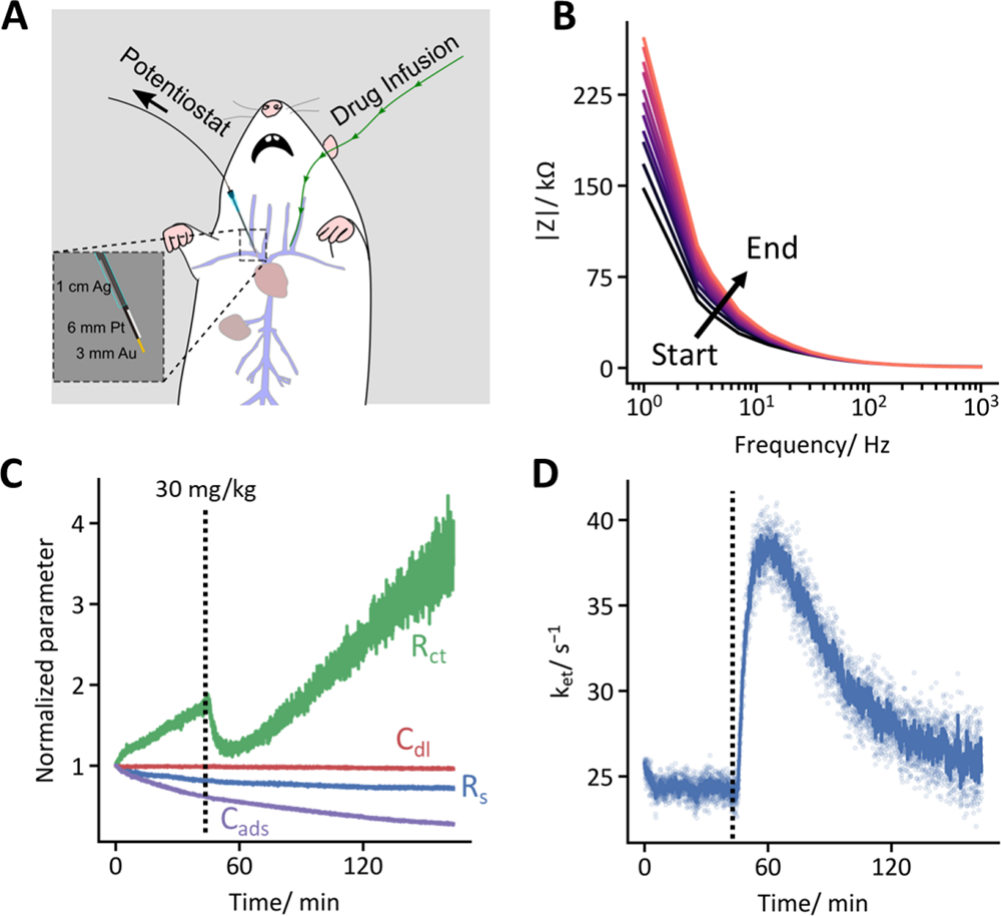
FFT-EIS supports high-frequency, real-time measurements in the living body. (A) In vivo EAB sensors are comprised of an aptamer-functionalized gold working electrode, a platinum counter electrode, and an Ag/AgCl reference electrode, each bound in heat-shrink tubing and inserted via a catheter into the right jugular vein of an anesthetized rat. (B) Using FFT-EIS, we measured impedance spectra every ∼1.8 s by applying the formal potential of methylene blue as the DC bias and the necessary multisine waveform as an AC perturbation. The magnitude of the impedance, |*Z*|, increased over the duration of the experiment, particularly at low frequencies. Presumably, this is due to fouling caused by nonspecific adsorption of proteins, cells, or small molecules to the sensor surface. (C) Fitting the spectra reveals that this drift is correlated to an immediate, steady increase in *R*_ct_ and a corresponding decrease in *C*_ads_. Upon injection of 30 mg/kg vancomycin, however, only *R*_ct_ is responsive. (D) Calculation of *k*_et_ reveals that this parameter is stable prior to drug infusion, indicating that the intrinsic electron transfer rate constant of the unbound state of the aptamer is unaffected by whatever is causing *R*_ct_ and *C*_ads_ to drift. Upon drug infusion, however, *k*_et_ rises suddenly, indicating a larger population of the target-bound state of the aptamer. After the infusion is concluded, *k*_et_ falls as the drug is excreted by the kidneys, and the unbound aptamer again dominates. Here, the raw data (light blue points) are smoothed using a 13-s rolling average (dark blue trace).

FFT-EIS interrogation of EAB sensors supports the rapid measurement of specific molecules in the living body. To demonstrate this, we bundled aptamer-modified gold wire working electrodes with platinum counter and silver–silver chloride reference electrodes in a 20-gauge catheter (Figure 3A) and surgically inserted the resulting three-electrode sensor into the right jugular vein of an anesthetized rat.^43^ We then measured full impedance spectra (containing 18 frequencies between 1 Hz and 1 kHz; Figure SI3) every 1.8 s for 2.5 h. Of note, the magnitude of the impedance increases over time in vivo (Figure 3B). This contrasts with the relatively unchanging impedance that we observed in whole blood in vitro (Figure SI4), suggesting that the mechanisms by which EAB sensors degrade may differ between the two conditions. Fitting the time-resolved spectra indicates that this change in impedance is associated with a steady increase in *R*_ct_ and a corresponding decrease in *C*_ads_ (Figure 3C). This presumably occurs due to the nonspecific adsorption of proteins and cells to the electrode and/or aptamer loss (although reductive stripping of aptamers should be minimized by the narrow potential window in EIS),^44^ which we expect will increase *R*_ct_ and decrease *C*_ads_ by reducing the number of methylene blue reporters that can access the electrode surface. In contrast, *k*_et_ does not drift (Figure 3D), indicating that whatever causes impedance to drift does not affect the electron transfer kinetics of the aptamers that remain electrochemically accessible. While this is perhaps surprising given the likelihood of electrode fouling occurring in vivo, this drift resistance agrees with the exceptional baseline stability (for >24 h in 37 °C whole blood) previously reported for phase-interrogated EAB sensors in vitro.^31^ Upon the infusion of 30 mg/kg vancomycin, however, *k*_et_ rapidly rises as the aptamer shifts to its target-bound conformation (Figure 3D). Following the end of the infusion, *k*_et_ returns to its baseline value, as the drug is removed from the plasma via the kidneys. This said, the signal-to-noise ratio does fall at later stages of the experiment, presumably due to the loss of aptamers.

**Figure 4:**
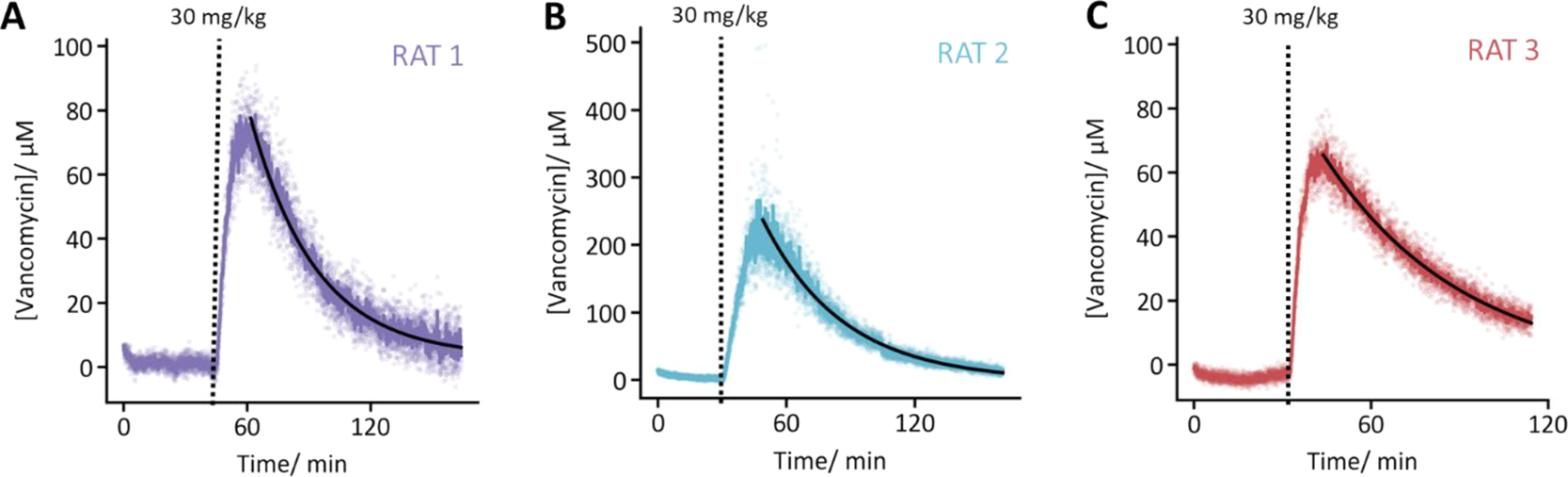
Using FFT-EIS to interrogate EAB sensors yields rapidly time-resolved molecular measurements in the living body. (A–C) Experiments in three separate animals confirm the absence of the drug preinfusion and show the expected concentration spike. Here, the raw data (light points; 1.8 s resolution) are smoothed using a seven-point (13 s) rolling average (darker trace). The antibiotic concentration decayed monoexponentially (fits shown as black traces) after each dosing, with time constants of (A) 33.1 ± 0.5 min, (B) 37.3 ± 0.7 min, and (C) 47.4 ± 1.3 min (95% confidence intervals). Of note, the initial *k*_et_ values of 25.1 ± 0.5, 27.0 ± 0.5, and 23.3 ± 0.4 s^–1^ are similar (errors reflect the standard deviation of the first one hundred data points) despite variable aptamer loadings (calculated from *C*_ads_ to be 1.08 ± 0.03, 0.61 ± 0.02, and 0.74 ± 0.02 pmol). Thus, *k*_et_ is relatively insensitive to small changes in the aptamer packing.

FFT-EIS interrogation of EAB sensors provides a highly time-resolved window into molecular physiology and pharmacokinetics (Figure 4A). To demonstrate this, we placed sensors into the jugular veins of three rats. Prior to drug infusion, we measured vancomycin concentrations fluctuating tightly around zero (mean ± one standard deviation = 1.1 ± 1.7 μM). While this is a marginally higher noise than seen for the same in vivo sensor when interrogated using square wave voltammetry (±1 μM),^43^ the present EIS technique allows for 7-fold faster data acquisition–application of a seven-point rolling average reduces our noise to just ±0.8 μM. Following infusion of the drug, its concentration is observed to rise to maxima of 70–250 μM before decreasing exponentially with a time constant of 32–47 min. The decay rates observed between three independent animals are similar to our previous observation and reflect each animal’s unique physiology,^8^ highlighting the benefit of using EAB sensors to individualize clinical dosing.

**Figure 5:**
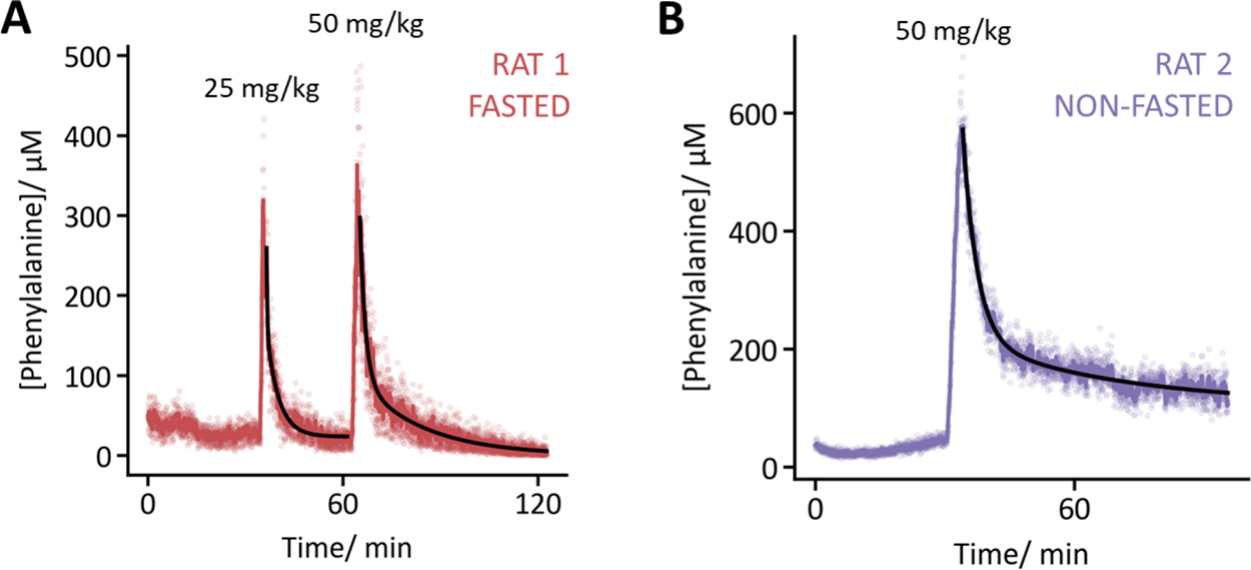
Using FFT-EIS to interrogate phenylalanine-detecting EAB sensors, we have monitored this metabolite in situ in the jugulars of live rats with 1.8 s resolution. (A) A fasted animal was infused with two sequential doses of phenylalanine. In both cases, the concentration of free phenylalanine in the blood quickly decayed back to the preinfusion baseline (41 ± 10, 31 ± 9, and 25 ± 10 μM before injection, after the first injection, and after the final injection, respectively). (B) In contrast, the return to the postinfusion baseline was slower in a nonfasted animal, which is consistent with previous reports regarding phenylalanine homeostasis.^11^ Here, the raw data (light points) are smoothed by using a 13-s rolling average (darker trace). Concentration decay transients were fit to a two-compartment (i.e., biexponential) model (black traces).

To demonstrate the general applicability of FFT-EIS as an EAB sensor interrogation technique, we next applied it to a sensor against the endogenous target phenylalanine (Figure 5). Specifically, after calibrating a phenylalanine-detecting EAB sensor^11^ in vitro (details on the sensor calibration can be found in the Supporting Information, Figure SI5), we used FFT-EIS interrogation to measure the molecule’s concentration in the jugular veins of anesthetized rats. Doing so, we observed baseline phenylalanine concentrations of 41 ± 10 μM (the latter reflects one standard deviation) in a fasted animal and 39 ± 6 μM in a nonfasted animal, values in line with previous reports.^11,45^ Upon two intravenous infusions of additional phenylalanine, we observed rapid rises to peak concentrations of 300 to 400 μM followed by rapid decays back to baseline in the fasted animal. Fitting the decay transients to the previously reported biexponential model of phenylalanine kinetics^11^ yielded time constants of τ_1_ = 0.2 ± 0.1 and τ_2_ = 3.8 ± 0.2 min for the first injection, and τ_1_ = 1.7 ± 0.1 and τ_2_ = 20 ± 2 min for the second (error bars are 95% confidence intervals), suggesting that the animal’s ability to rapidly store additional phenylalanine may have been saturated after the first challenge. In a nonfasted animal, in contrast, we measured a higher peak concentration (∼600 μM) and a slower decay (τ_1_ = 3.3 ± 0.1 and τ_2_ = 35 ± 7 min) to a higher, slowly decaying baseline following phenylalanine challenge, discrepancies that align with previous, in vivo measurements of phenylalanine kinetics in fasted and nonfasted rats.^11^

Looking forward, we believe it may prove possible to improve EIS-interrogated EAB sensors still further, which may be of value in monitoring rapid physiological processes such as neurotransmitter release^46^ and the pharmacodynamics of psychoactive drugs.^47,48^ For example, using a thinner monolayer,^49^ a shorter DNA strand,^50^ or a more rapidly electron-transferring redox reporter would increase *k*_et_, thus raising the lowest frequency that needs to be sampled and, with that, further improving time resolution. Likewise, future studies should focus on longer duration experiments and experiments in awake animals which, while rendered difficult due to animal welfare concerns,^7^ are necessary for improving sensor longevity and translating EAB technology to the clinic.

## Conclusions

We have established FFT-EIS as a rapid, reliable, calibration-free method of interrogating EAB sensors, both in vitro and in vivo. Specifically, we demonstrated the ability of FFT-EIS to measure the electron transfer rate associated with EAB sensors and used this to determine the concentration of their molecular targets with a time resolution of just 1.8 s. Because this approach uses *k*_et_ as a means of monitoring target concentration, rather than absolute current, it is independent of both sensor-to-sensor fabrication variation and the drift arising due to fouling in biological fluids, rendering the technique suitable for performing calibration-free in vivo measurements. In support of this, we used vancomycin- and phenylalanine-detecting EAB sensors to successfully monitor plasma concentrations of these targets in the veins of live animals, with time resolution of better than 2 s and without requiring the calibration of each, individual sensor.^11^ When combined with the modularity of aptamers, the benefits associated with impedimetric interrogation of EAB sensors could improve our understanding of pharmacokinetics and metabolism and play an important role in the future of personalized medicine.

## Experimental Section

### Materials

In-vitro sensors were made using 0.2 mm diameter gold wire (99.99%, Thermo Fisher) insulated with polyolefin heat-shrink tubing (0.05″,0.017″, 0.007″, McMaster-Carr). For in vitro tests, we used a commercial Ag/AgCl(s) reference electrode and a commercial platinum wire counter electrode (CH Instruments Inc.). Intravenous sensors used for in vivo measurements were made using 0.2 mm diameter gold wire, 0.005 in. diameter platinum wire and 0.005 in. diameter silver wire (all 99.99% purity, A–M Systems). The insulation used for these sensors was polytetrafluoroethylene heat-shrink (HS Sub-Lite-Wall, 0.02, 0.005, 0.003 ± 0.001 in, black, Zeus Inc.) Sodium hydroxide, 6-mercapto-1-hexanol, Tris (2-carboxyethyl) phosphine, sulfuric acid, phenylalanine, and the phenylalanine assay kit were obtained from Sigma-Aldrich. Phosphate buffered saline (PBS) was diluted from a 20× stock purchased from Santa Cruz Biotechnologies. Vancomycin–HCl was purchased from VWR. Methylene blue- and HO–C_6_S–S–C_6_-modified DNA sequences were purchased from Integrated DNA Technologies (Coralville, Iowa); their sequences are listed in Table 1. We chose these sequences due to (a) their reliability in previous EAB studies, (b) the fact that the K_D_s of these aptamers overlap with the physiologically relevant ranges of their target molecules, and (c) that both yield stable, high signal gain EAB signals when interrogated using SWV.^11,39^

**Table 1:** DNA Sequences Employed in This Study

name	sequence and modifications
vancomycin	HS-C_6_–CGAGGGTACCGCAATAGTACTTATTGTTCGCCTATTGTGGGTCGG-MB
phenylalanine	HS-C_6_–CG ACC GCG TTT CCC AAG AAA GCA AGT ATT GGT TGG TCG-MB

### Sensor Fabrication

In vitro sensors were made by shrink wrapping gold wire with polyolefin and leaving 3 mm of the wire exposed. These sensors were made ahead of time and required no additional steps prior to electrochemical cleaning. We constructed our intravenous sensors as previously reported.^43^ Briefly, they are made using gold, platinum, and silver wires. These wires were individually insulated with polytetrafluoroethylene heat-shrink and bundled together in a staggered manner with the gold wire at the bottom, followed by the platinum and then the silver wire. The exposed lengths of each wire were 3, 6, and 1 cm, respectively. Once bundled together, the intravenous, three-electrode sensors were immersed overnight in household bleach (Clorox, sodium hypochlorite 7.5%) to chlorinate the silver electrode. The three electrodes were subsequently rinsed with Millipore water prior to electrochemical cleaning.

Prior to aptamer deposition, we electrochemically cleaned the gold working electrode in NaOH followed by roughening in H_2_SO_4_ by using a CH1040C potentiostat. The cleaning involved cycling the potential between −1.0 V and −2 at 2 V/s 1000 times while the electrodes were immersed in 0.5 M NaOH.^51^ This was followed by roughening in 0.5 M H_2_SO_4_ with the application of 20 ms pulses at 0 and 2.2 V 32,000 times as previously done to increase the electrode’s microscopic surface area.^52^ The electrodes were subsequently analyzed by cyclic voltammetry in 0.5 M H_2_SO_4_ (between 1.5 and −0.35 at 1 V/s) to determine their electroactive surface area.^53^ Intravenous sensors that were to be used in vivo were inserted into a 20G catheter (Becton, Dickinson, and Company) at this point.

To functionalize the working electrode, we first reduced the disulfide bond in the stock alkanethiol-and-methylene-blue-modified aptamer by combining 14 μL of 10 mM tris (2-carboxyethyl) phosphine with 2 μL of 100 μM DNA for 1 h in the dark. We then rinsed the electrochemically cleaned and roughened gold electrodes with Millipore water and immersed them for 1 h in 500 nM reduced DNA in PBS. The electrodes were then transferred to a 10 mM solution of 6-mercapto-1-hexanol in PBS and stored overnight before use.

### Electrochemical Measurements

All electrochemical measurements were carried out using a three-electrode setup. In our in vitro experiments, we employed a Ag/AgCl (saturated KCl, CH Instruments Inc.) reference electrode and a platinum wire counter electrode (CH Instruments Inc.). In vivo, we used a silver wire coated with silver chloride (as described above) as our reference electrode and a platinum wire counter electrode.

All electrochemical measurements were performed using an Autolab PGStat128N (Metrohm). The potentiostat was configured in “high stability mode” with a current range of ±1 μA, which affects the filter characteristics. For FFT-EIS measurements, the multisin waveform was generated by a DG812 arbitrary waveform generator (Rigol Technologies) and fed into the potentiostat’s external voltage input using a BNC connection. Our voltage waveform consisted of a superposition of 18 sine waves at logarithmically spaced frequencies ranging from 1 Hz to 1 kHz. The amplitude and phase of each sinusoidal oscillation were optimized for maximum signal-to-noise as discussed in the Supporting Information,^34,37^ and the summed waveform was scaled to have a peak-to-peak amplitude of 25 mV. The potentiostat’s native software (NOVA) was used to set the DC bias–the formal potential of methylene blue, as measured by cyclic voltammetry–on top of the AC perturbation. Voltage and current were recorded at 70 kHz by using an SDS1202X-E oscilloscope (Siglent Technologies). After each oscilloscope frame (1.4 s) was collected, the current and voltage data were transferred to the host computer, Fourier-transformed, saved, and displayed on a GUI for real-time monitoring. Data recording and processing were controlled by a custom Python program. Further details on the chosen waveform and artifact correction are described in Supporting Information (Table SI1, Figures SI3 and SI6).

We fit impedance spectra to the equivalent circuit model by using MEISP 3.0 (Kumho Petrochemical Co. Ltd.) after each experiment. As discussed in the main text, the adsorption pseudocapacitance *C*_ads_ was modeled as a constant phase element, given by eq 2 (i is the imaginary number, ω is frequency, and n is the constant phase parameter). The parameter n was fixed at 0.84 for all fits to improve the consistency in the fitted *C*_ads_ values.
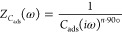
5

### In Vivo Measurements

All in vivo experiments were performed in male Sprague–Dawley rats (4–5 months old, Charles River Laboratories of Santa Cruz, CA). The rats weighed between 350 and 500 g and were pair-housed in a standard light cycle room (12:12 regular light cycle with lights on at 8AM). They were allowed ad libitum access to food and water, and the Institutional Animal Care and Use Committee (IACUC) of the University of California at Santa Barbara approved our experimental protocol which adhered to the guidelines given by the NIH Guide for Care and Use of Laboratory Animals (eighth edition, National Academy Press, 2011).

Prior to the measurement, we anesthetized the rats using 4% isofluorane in a Plexiglas anesthesia chamber. Anesthesia was then maintained via a nose cone for the entire duration of the experiment at a level of 2–2.5% isofluorane. The neck was shaved, and bilateral incisions were made in order to surgically isolate the left and right jugular veins. After isolation, each vein was tied off using sterile 6–0 silk sutures (Fine Science Tools, Foster City, CA) A small incision was then made in each vein using spring-loaded microscissors that allowed us to insert the sensor-containing catheter into the right jugular vein and an infusion line into the left jugular vein. Both the sensor and drug infusion catheter were anchored in place using two sterile 6–0 silk sutures (Fine Science Tools, Foster City, CA). Prior to the measurement, the wires in the sensor were adjusted such that the counter and working electrode were exposed outside of the 20G catheter into the vein as previously described,^43^ and we infused 30 units of heparin through the infusion line immediately after insertion of the sensor and prior to any recordings. To intravenously dose the rats at 30 mg/kg, we injected a precalculated volume of 0.05 M vancomycin solution using a syringe pump (KD Scientific) as previously described.^7^

## Supporting Information Available

Multi-frequency waveform table; cyclic voltammograms; k_et_ versus sensor size; multi-sin waveform; Bode |Z| plots for titration; phenylalanine calibration; and filter correction (PDF)
