# Supplementary material for: Calibration-Free, Seconds-Resolved In Vivo Molecular Measurements using Fourier-Transform Impedance Spectroscopy Interrogation of Electrochemical Aptamer Sensors
Source: ACS Sens. 2023 Aug 16;8(8):3051–9. doi: 10.1021/acssensors.3c00632 (PMC10463274; doi:10.1021/acssensors.3c00632)
Supplement: Supplementary file 1 — se3c00632_si_001.pdf [file se3c00632_si_001.pdf]

Supporting Information: Calibration-free, seconds-resolved in vivo molecular measurements using Fourier-transform impedance spectroscopy interrogation of electrochemical aptamer sensors

Brian Roehrich,[1,†] Kaylyn K. Leung,[1,4,†] Julian Gerson,[2,4] Tod E. Kippin,[2,3] Kevin W. Plaxco[1,4] and Lior Sepunaru[1*]

[1]Department of Chemistry and Biochemistry, University of California Santa Barbara, Santa Barbara, CA 93106, USA.

[2]Department of Psychological and Brain Sciences, University of California, Santa Barbara.

[3]Department of Molecular Cellular and Developmental Biology, University of California, Santa Barbara.

[4]Center for Bioengineering, University of California Santa Barbara, Santa Barbara, CA 93106, USA.

† B.R. and K.K.L contributed equally.

*Corresponding author. Email: sepunaru@ucsb.edu

**Table of Contents:**

1. Multi-frequency waveform table
2. Cyclic voltammograms
3. $k_{et}$ versus sensor size
4. Multi-sin waveform
5. Bode |Z| plots for titration
6. Phenylalanine calibration
7. Filter correction

1. Multi-frequency waveform

Table SI1. Frequencies, amplitudes, and phases used to construct the multi-frequency perturbation waveform.

| **Frequency/ Hz** | **Normalized Amplitude** | **Phase/ °** |
|---|---|---|
| 1 | 1.00 | 4 |
| 3 | 0.64 | -27 |
| 4 | 0.58 | 151 |
| 7 | 0.48 | 93 |
| 10 | 0.43 | 6 |
| 13 | 0.40 | 180 |
| 21 | 0.34 | 187 |
| 31 | 0.29 | 182 |
| 44 | 0.26 | 26 |
| 64 | 0.22 | 175 |
| 90 | 0.19 | 54 |
| 110 | 0.17 | 143 |
| 170 | 0.14 | 4 |
| 250 | 0.12 | 136 |
| 370 | 0.10 | 250 |
| 520 | 0.08 | 129 |
| 750 | 0.07 | 12 |
| 1000 | 0.06 | 164 |

2. Sensor cyclic voltammograms

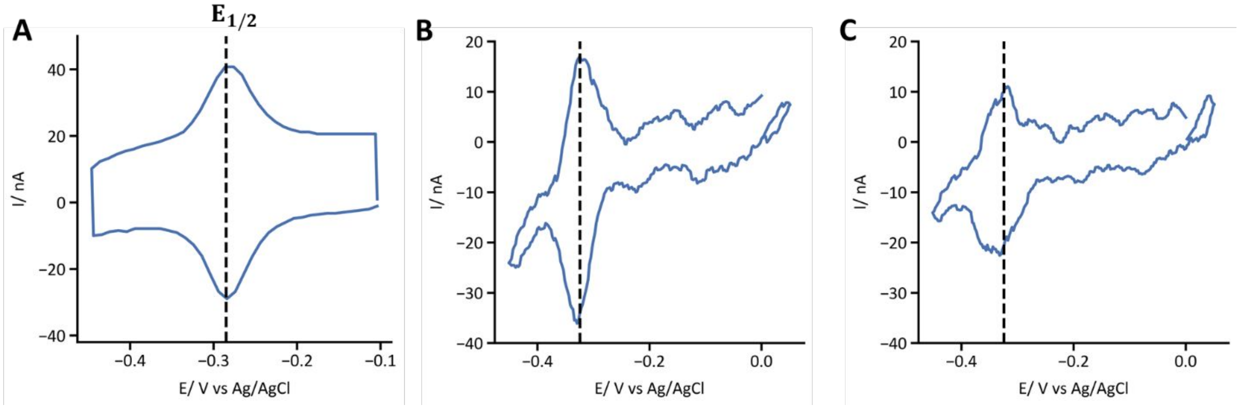


Figure SI1. (A) Cyclic voltammogram recorded from a vancomycin-detecting EAB sensor in 1X PBS plus 2 mM $MgCl_2$. The voltammogram was recorded at 25°C with a scan rate of 100 mV/s. The half wave potential (the mean voltage between the reductive and oxidative peak potentials), $E_{1/2}$, was determined to be -285 mV versus Ag/AgCl. We recorded cyclic voltammograms prior to all EIS experiments in order to determine $E_{1/2}$, which was applied as the DC bias during EIS. (B) In vivo, $E_{1/2}$ (black dashed line) is -324 mV; this difference arises from the use of an anodized silver wire as the reference electrode rather than the fritted, single junction reference electrode used *in vitro*. (C) Shown is a cyclic voltammogram recorded in vivo at the end of a vancomycin dosing experiment. While the peak area is reduced (presumably due to monolayer loss),[1] $E_{1/2}$ remains at -324 mV.

3. $k_{et}$ versus sensor size

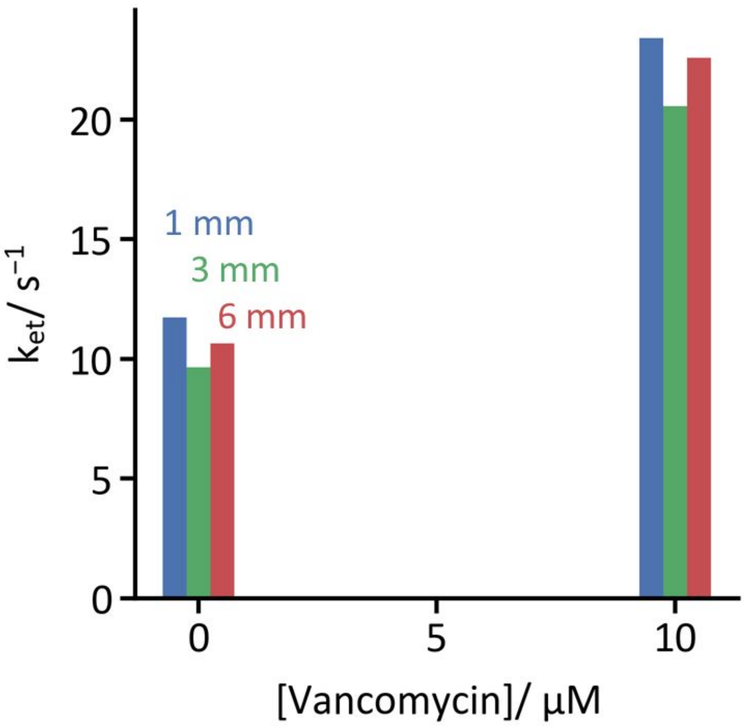


Figure SI2. Electron transfer rate constants ($k_{et}$) recorded for vancomycin-sensing electrodes immersed in phosphate-buffered saline at 25 ℃. The electrodes were fabricated with variable lengths of exposed gold wire (1, 3, and 6 mm) before they were subjected to the roughening and aptamer deposition procedures outlined in the experimental section. Both in the absence of vancomycin and when challenged with 10 μM of vancomycin, the recorded $k_{et}$ values are tightly clustered and there is no systematic trend with electrode size, verifying the theoretical prediction that $k_{et}$ is independent of electrode surface area. We note that these $k_{et}$ values are lower than those presented in Figure 2D because this experiment was performed at 25 ℃.[2]

4. Multi-sin waveform

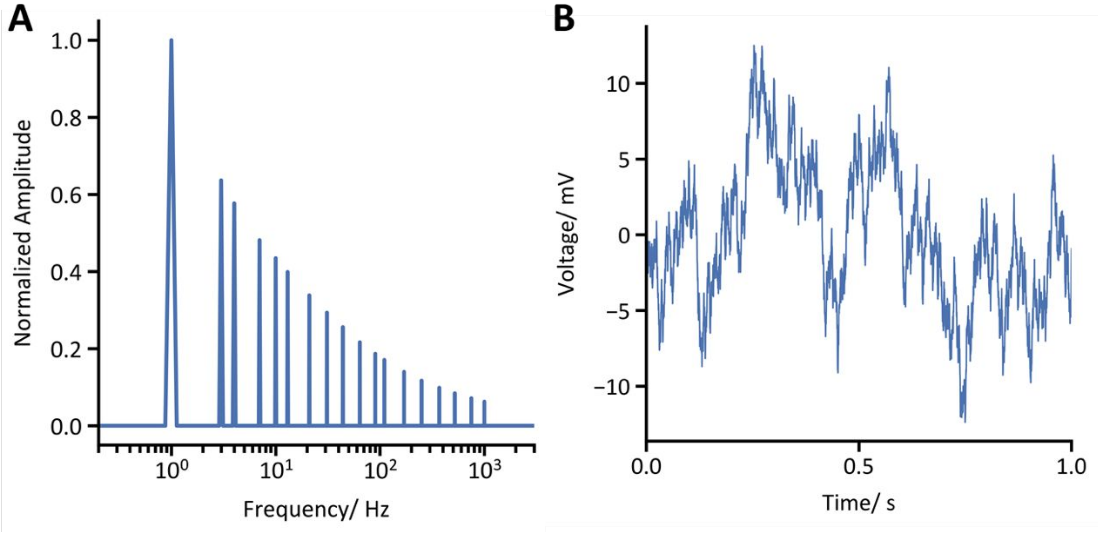


Figure SI3. (A) Frequency- and (B) time-domain representations of the multi-sine EIS waveform we employed.

We based our multi-sine waveform on the optimizations reported by Popkirov and Schindler,[3] and on our previous report.[4] A set of 18 logarithmically-spaced frequencies were chosen which were integer multiples of the fundamental frequency (1 Hz), avoiding any second harmonics. Phases were chosen in order to minimize constructive interference as previously described.[3] Amplitudes were set to create a similar current output at every frequency (i.e., $V(\omega) \propto |Z|(\omega)$). This strategy significantly increases signal-to-noise by applying higher voltage amplitudes at low frequencies, where (in an electrochemical cell) current is typically lower than at high frequencies. Using the frequencies $f$, amplitudes $a$, and phases $\phi$ listed (Table S1), the waveform was digitally synthesized as:

$$v(t) = \sum_j a_j \sin\left(2\pi f_j t + \phi_j\right) \tag{S1}$$

The optimized waveform used in this study is shown in the frequency domain in Figure SI3A and in the time domain in Figure SI3B. In the time domain, the peak-to-peak amplitude of the summed waveform was set to be 25 mV. The digital waveform was saved to a Rigol DG812 arbitrary waveform generator, which output the waveform at 100 kHz into the voltage input of the Autolab PGStat128N.

5. Bode |Z| plot for titration

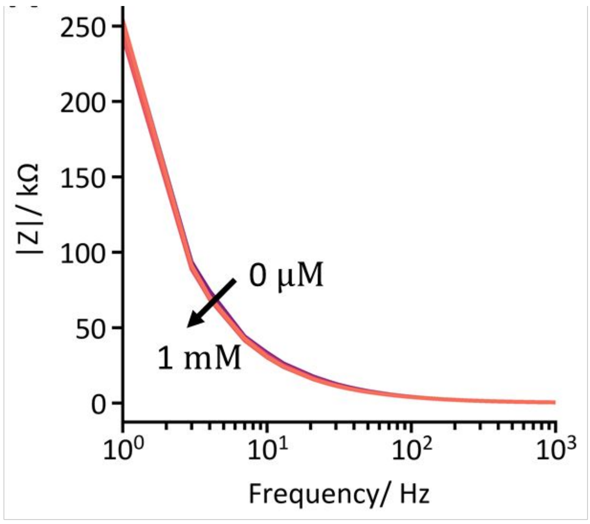


Figure SI4. Bode |Z| plots collected from a vancomycin-detecting EAB immersed in whole bovine blood at 37℃ and challenged with increasing concentrations of vancomycin (the same 18 concentrations as Figure 1D and 2B in the main text; here, all curves overlap). The modulus of the impedance does not change significantly at any frequency as a function of target concentration, despite the observed phase shifts (Figure 2A).

6. Phenylalanine calibration

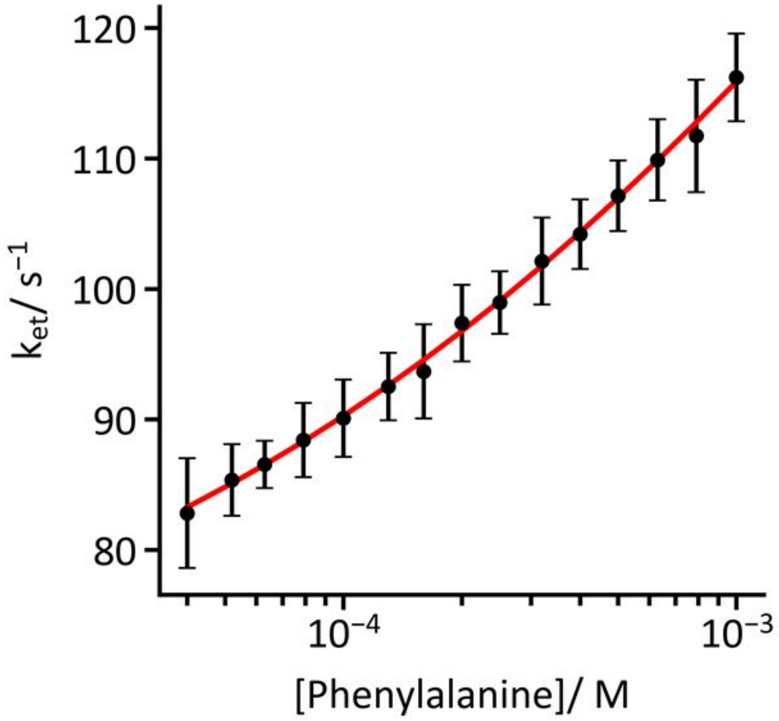


Figure SI5. The binding curve of a phenylalanine-detecting EAB sensor interrogated using EIS can be used to determine phenylalanine concentration from $k_{et}$. To record this curve, we immersed the sensor in whole, freshly-collected rat's blood at 37℃. The endogenous concentration of phenylalanine in the blood (52 μM) was determined using a fluorescent assay kit. Aliquots of phenylalanine dissolved in PBS-BSA were then added to the blood to increase the phenylalanine concentration. Values of $k_{et}$ (black points, error bars represent the standard deviation across four independently fabricated and tested sensors) fall on a Langmuir isotherm,

$$k_{et} = k_{et,0} + (k_{et,\,max} - k_{et,0})\frac{[Phe]^n}{K_D^n + [Phe]^n} \tag{S2}$$

where $k_{et,0}$ is the value of $k_{et}$ in the absence of phenylalanine, $k_{et,max}$ is the value of $k_{et}$ at saturating phenylalanine, $K_D$ is the dissociation constant, and n is the Hill coefficient.[2] Fitting to this equation yielded $k_{et,0}$ = 62.9 $s^{-1}$, $k_{et,max}$ = 225.8 $s^{-1}$, $K_D$ = 6.89 mM, and n = 0.38.

7. Filter correction

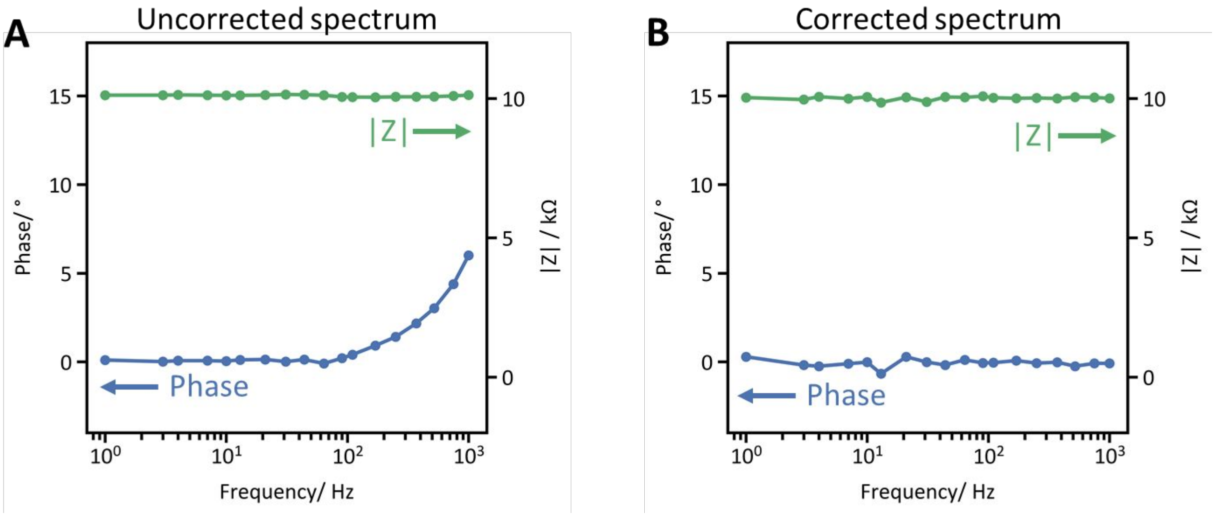


Figure SI6. Phase (blue) and modulus (green) Bode plots of a 10 kΩ resistor. (A) The spectrum without filter corrections. |Z| is the expected 10 kΩ from 1 Hz to 1 kHz, but the phase is nonzero at high frequencies. (B) After correction, phase is zero across the frequency spectrum.

The low-pass current filter applied by the potentiostat may affect the measured impedance spectra, and should be corrected for. To do this, we measured the impedance spectrum of a 10 kΩ resistor (Figure SI6A). While we expect a constant impedance of 10 kΩ and a constant phase of 0° for this resistor, we measure a phase shift that reaches 7° at 1 kHz. This is caused by the tail of the low-pass filter, which cannot be directly controlled on the potentiostat used in this study but which is affected by the choice of current range. Subsequently, impedance spectra were corrected using this reference spectrum as shown in Equations S3 and S4:

$$|Z|(\omega) = \frac{|Z|_{measured}(\omega)}{|Z|_{ref}(\omega)} \quad \text{(S3)}$$

$$\phi(\omega) = \phi_{measured}(\omega) - \phi_{ref}(\omega) \quad \text{(S4)}$$

Re-recording the impedance spectrum of the same 10 kΩ resistor and applying this correction procedure yielded the expected 10 kΩ |Z|, 0° phase across the entire spectrum (Figure SI6B).

**References**


(1) Leung, K. K.; Downs, A. M.; Ortega, G.; Kurnik, M.; Plaxco, K. W. Elucidating the Mechanisms Underlying the Signal Drift of Electrochemical Aptamer-Based Sensors in Whole Blood. *ACS Sensors* **2021**, *6* (9), 3340–3347.

(2) Downs, A. M.; Gerson, J.; Leung, K. K.; Honeywell, K. M.; Kippin, T.; Plaxco, K. W. Improved Calibration of Electrochemical Aptamer-Based Sensors. *Sci. Rep.* **2022**, *12* (1), 1–10.

(3) Popkirov, G. S.; Schindler, R. N. Optimization of the Perturbation Signal for Electrochemical Impedance Spectroscopy in the Time Domain. *Rev. Sci. Instrum.* **1993**, *64* (11), 3111–3115.

(4) Roehrich, B.; Liu, E. Z.; Silverstein, R.; Sepunaru, L. Detection and Characterization of Single Particles by Electrochemical Impedance Spectroscopy. *J. Phys. Chem. Lett.* **2021**, *12* (40), 9748–9753.
